# Relationship between central and peripheral fatty acids in humans

**DOI:** 10.1186/1476-511X-12-79

**Published:** 2013-05-28

**Authors:** Jade Guest, Manohar Garg, Ayse Bilgin, Ross Grant

**Affiliations:** 1 School of Medical Sciences, University of New South Wales, Wallace Wurth building, office #203, Sydney, NSW 2052, Australia; 2 Australasian Research Institute, Sydney Adventist Hospital, Sydney, NSW, Australia; 3 Nutraceuticals Research Group, School of Biomedical Sciences & Pharmacy, University of Newcastle, Newcastle, NSW, Australia; 4 Department of Statistics, Macquarie University, North Ryde, NSW, Australia; 5 Sydney Adventist Hospital Clinical School, University of Sydney, Sydney, NSW, Australia

**Keywords:** Fatty acids, Polyunsaturated fatty acids, Brain, Whole blood, Cerebrospinal fluid, Blood brain barrier, Omega-3, Omega-6

## Abstract

**Background:**

In recent years the physiological and pathological importance of fatty acids in both the periphery and central nervous system (CNS) has become increasingly apparent. However surprisingly limited research has been conducted comparing the fatty acid composition of central and peripheral lipid stores.

**Methods:**

The present study compared the distribution of polyunsaturated (PUFA), as well as specific saturated (SFA) and monounsaturated (MUFA) fatty acids in the whole blood and cerebrospinal fluid (CSF) of humans. Gas chromatography with flame ionization detection was used to determine the fatty acid profiles of twenty-eight matched CSF and whole blood samples. Multiple linear regression modeling, controlling for age, was used to identify significant relationships.

**Results:**

A significant positive relationship was seen between whole blood total omega-3 fatty acids and the CSF omega-3 subfractions, docosapentaenoic acid (DPA) (P = 0.019) and docosahexaenoic acid (DHA) (P = 0.015). A direct association was also observed between the whole blood and CSF omega-6 PUFA, arachidonic acid (AA) (P = 0.045). Interestingly an inverse association between central and peripheral oleic acid was also found (P = 0.045).

**Conclusions:**

These findings indicate a relationship between central and peripheral fatty acids of varying degrees of unsaturation and chain length and support the view that some systemic fatty acids are likely to cross the human blood brain barrier (BBB) and thereby influence central fatty acid concentrations.

## Background

Nutritional supply of essential fatty acids is considered important for numerous physiological functions at every stage of human life. At sufficient levels of incorporation, fatty acids influence a number of cellular functions including cell membrane fluidity [1], membrane protein-mediated responses [2], eicosanoid generation [3], gene expression [4], and cell signaling [5]. Through these mechanisms, fatty acids influence cell and tissue physiology, and the way cells and tissues respond to external signals in both the periphery and CNS.

The unique lipid profile of the CNS has been recognized for a number of decades. Of all organs in the human body, excluding adipose tissue, the nervous system has the highest lipid content. The dry weight of an adult brain is approximately fifty-five percent lipid, thirty-five percent of which is accounted for by PUFA’s. Of these, docosahexaenoic acid (DHA; C22:6n-3) and arachidonic acid (AA; C20:4n-6), respectively, are present in the highest concentrations [6]. In comparison alpha-linolenic acid (ALA; C18:3n-3), eicosapentaenoic acid (EPA; C20:5n-3), and docosapentaenoic acid (DPA; 22:5n-3) comprise only one percent of total brain fatty acids [7]. Since these early observations many functions of brain PUFA’s, including their role in neurogenesis, cell survival, signal transduction, and neuroinflammation have been identified [8-11]. Furthermore alterations in brain PUFA metabolism have been linked to various neurological disorders including major depression and Alzheimer’s disease [12,13].

A comparatively small body of research has focused on the function of other fatty acid species within the brain. Evidence suggests that in appropriate quantities, both SFA and their derivatives are required for brain health. In a recent study using human frontal cortex tissue Martín et al. demonstrated that lipid rafts, membrane structures intimately associated with cell signalling, are predominately composed of the SFA’s palmitic (C16:0) and stearic (C18:0) acid [14]. Oleic acid (C18:1n-9), formed from the desaturation of stearic acid, has been shown to promote axonogenesis in the striatum during brain development [15], and is used as a cerebral energy source when glucose availability diminishes [16].

Even though the important role of fatty acids within the periphery and CNS has been firmly established, the mechanisms by which fatty acids enter the brain are still not fully understood. The brain is protected by the BBB that forms a cellular interface between the blood and the brains extracellular matrix. In order to enter the brain, fatty acids must move across both luminal and abluminal membranes of the BBB endothelial cells, and then cross the plasma membrane of neural cells. It is currently thought that fatty acids cross the BBB via either specific fatty acid transporter proteins, passive diffusion using a ‘flip flop’ mechanism or a combination of both [17-19].

While the physiological and pathological importance of fatty acids in both the periphery and CNS has become increasingly apparent, surprisingly limited research has been conducted comparing the fatty acid composition of the CNS with blood, still fewer studies have been conducted in humans. Does the content of fatty acids in human blood reflect the CNS lipid profile, and if so, to what extent? As the development of some neurodegenerative disorders have been linked to alterations in both brain and red blood cell fatty acid proportions [12,13], and because rodents are not always reliable as preclinical models for human disease, the present study sought to determine the distribution of PUFA’s, as well as specific SFA and MUFA, in matched human whole blood and CSF samples.

## Results

The distribution of various fatty acids in the CSF and whole blood are presented in Table 1, expressed as a percentage of total fatty acids.

**Table 1:** Comparison between whole blood and cerebrospinal fluid fatty acids levels

**Fatty acid**		**Whole blood**^**a**^**(%)**			**CSF**^**a**^**(%)**		
**C:Dn-x**	**Common name**	**Mean (±SE)**	**Min**	**Max**	**Mean (±SE)**	**Min**	**Max**
C16:0	palmitic acid	23.37 (0.43)	18.77	27.68	27.88 (0.76)	14.77	55.55
C16:1n-7	palmitoleic acid	2.56 (0.15)	0.75	4.43	2.71 (0.18)	0.89	7.24
C18:0	stearic acid	8.68 (0.28)	6.59	11.55	18.70 (0.78)	9.79	41.11
C18:1n-7	vaccenic acid	2.22 (0.08)	1.48	3.31	3.76 (0.17)	0.00	7.61
C18:1n-9	oleic acid^c^	21.21 (0.50)	15.8	26.17	25.48 (0.61)	11.74	33.94
C18:2n-6	linoleic acid	20.40 (0.53)	13.33	26.04	7.01 (0.33)	2.87	17.47
C18:3n-3	alpha-linolenic acid	0.74 (0.04)	0.46	1.42	2.54 (0.26)	0.59	16.14
C18:3n-6	gamma-linolenic acid	1.52 (0.06)	0.98	2.06	ND	-	-
C20:0	arachidic acid	0.26 (0.02)	0.12	0.51	ND	-	-
C20:1n-9	eicosenoic acid	0.24 (0.01)	0.17	0.37	1.21 (0.14)	0.00	6.14
C20:2n-6	eicosadienoic acid	1.17 (0.08)	0.30	2.01	ND	-	-
C20:3n-6	dihomo-gamma-linolenic acid	1.73 (0.05)	1.07	2.31	1.02 (0.12)	0.00	4.84
C20:4n-6	arachidonic acid^b^	8.00 (0.38)	4.78	12.47	3.47 (0.20)	0.99	9.00
C20:5n-3	eicosapentaenoic acid	2.01 (0.15)	1.04	3.87	1.87 (0.28)	0.00	10.66
C22:5n-3	docosapentaenoic acid^c^	1.98 (0.10)	1.06	3.17	2.94 (0.18)	0.84	8.46
C22:6n-3	docosahexaenoic acid^c^	3.91 (0.17)	2.54	5.92	1.41 (0.12)	0.00	5.70
	Total SFA	32.31 (0.28)	29.06	35.20	46.58 (1.27)	24.57	75.94
	Total MUFA^b^	26.26 (0.57)	18.92	31.71	33.16 (0.69)	14.55	41.21
	Total omega-3 PUFA^c^	8.64 (0.33)	6.05	12.66	8.76 (0.55)	2.65	28.52
	Total omega-6 PUFA	32.80 (0.53)	25.63	38.79	11.50 (0.49)	4.85	23.53
	Ratio omega-6:omega-3	3.92 (0.13)	2.73	5.54	1.47 (0.07)	0.41	3.45

Fatty acids are expressed as a percentage of total fatty acids.

^a^n = 28, ^b^P < 0.05 for comparison between respective whole blood and CSF fatty acid concentrations using multiple linear regression.

^c^P < 0.05 for comparison between whole blood total omega-3 PUFA and CSF DPA (C22:5n-3) and DHA (C22:6n-3) using multiple linear regression.

*ND* not detected.

### CSF and whole blood PUFA’s

**Figure 1:**
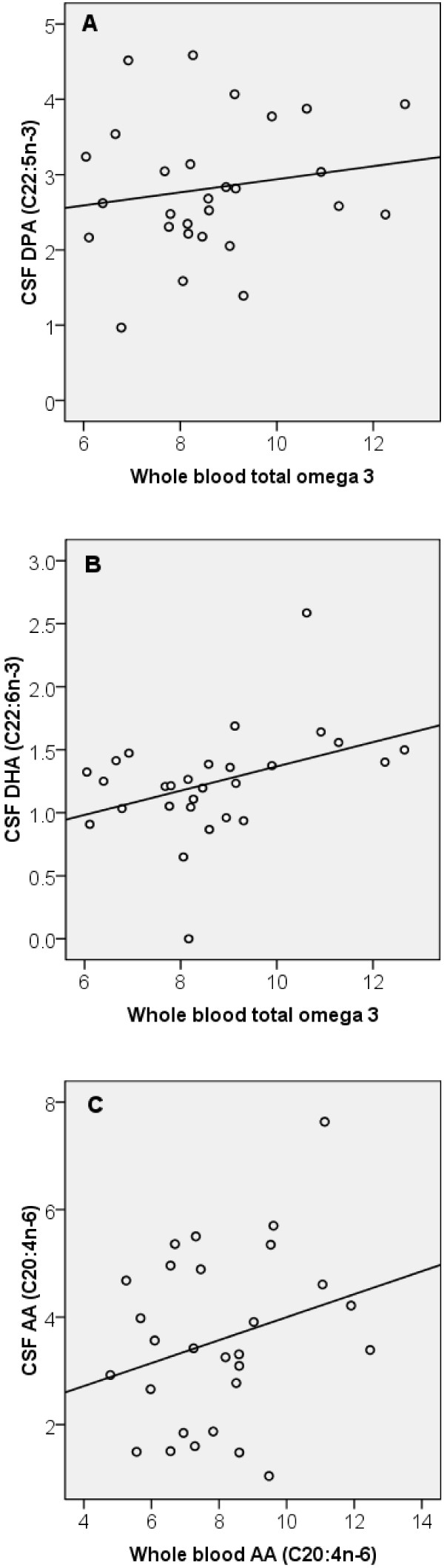
**Relationship between peripheral and central PUFA.** (**A**) A significant positive association was observed between whole blood total omega-3 and the CSF omega 3 subfraction DPA (P = 0.019, n = 28). (**B**) Likewise a significant positive association was observed between whole blood total omega-3 and the CSF omega 3 subfraction DHA (P = 0.015, n = 28). (**C**) A positive association was observed between whole blood and CSF AA (P = 0.045, n = 28). Comparisons were made using multiple linear regression controlling for age.

No association was observed for individual omega-3 PUFA (ALA or EPA or DPA or DHA) between whole blood and CSF. However a significant positive relationship was seen between whole blood total omega-3 fatty acids and the CSF omega-3 subfractions i.e. DPA (C22:5n-3) (P = 0.019, n = 28) and DHA (C22:6n-3) (P = 0.015, n = 28) (Figure 1). A one unit increase in whole blood total omega-3 fatty acid was associated with a 0.275 unit increase in CSF DPA. Similarly a one unit increase in whole blood total omega-3 fatty acid inferred a 0.143 unit increase in CSF DHA.

A positive relationship was also observed between the whole blood and CSF omega-6 PUFA AA (C20:4n-6) (P = 0.045, n = 28) (Figure 1). A one unit increase in whole blood AA was associated with a 0.865 unit increase in CSF AA. No associations were observed for other omega-6 PUFA.

### CSF and whole blood MUFA’s

**Figure 2:**
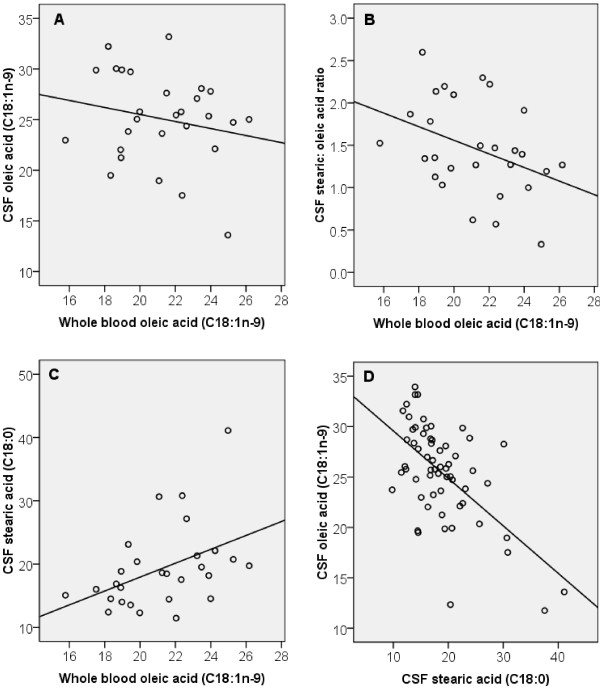
**Relationship between peripheral and central SFA and MUFA.** (**A**) A significant inverse association was observed between whole blood and CSF oleic acid (P = 0.026, n = 28). (**B**) A significant inverse association was observed between whole blood oleic acid and the CSF stearic: oleic acid ratio (P = 0.002, n = 28) (**C**) A significant positive association was observed between whole blood oleic and CSF stearic acid (P = 0.004, n = 28). (**D**) A significant inverse association was observed between CSF stearic and oleic acid (P = 0.000, n = 28). Comparisons were made using multiple linear regression controlling for age.

In this cohort a significant inverse relationship was observed between whole blood and CSF oleic acid (C18:1n-9) (P = 0.026, n = 28) (Figure 2). A one unit increase in whole blood oleic acid was associated with a 0.836 unit decrease in CSF oleic acid. Consistent with this, a significant inverse relationship was also detected between whole blood and CSF total MUFA (P = 0.013, n = 28). No associations were observed for other individual MUFA species.

In order to explain the unexpected inverse relationship observed between whole blood and CSF oleic acid, the data was further analyzed to determine if there was an association between whole blood oleic and the CSF stearic: oleic acid ratio, a putative index of Δ9-desaturase activity. As predicted, due to the ability of oleic acid to inhibit Δ9-desaturase, a one unit increase in whole blood oleic acid was associated with a 0.141 unit decrease in the CSF stearic: oleic acid ratio (P = 0.002, n = 28). Correspondingly a one unit increase in whole blood oleic acid was associated with a 1.587 unit increase in CSF stearic acid (P = 0.004, n = 28) and a one unit increase in CSF stearic acid was associated with a 0.466 unit decrease in CSF oleic acid (P = 0.000, n = 28) (Figure 2).

### CSF and whole blood SFA’s

No associations were observed between whole blood and CSF SFA species.

## Discussion

While a significant body of work has focused on the function of fatty acids in both the periphery and CNS, limited research has been conducted on the compartmentalization of fatty acids between blood and cerebrospinal fluid. To our knowledge this is the first study to compare the distribution of PUFA, as well as specific SFA and MUFA, in the whole blood and CSF of humans. In this cohort we found that whole blood total omega-3 fatty acid levels were associated with the CSF omega-3 PUFA’s, DPA and DHA. This is consistent with a recent report by Jumpertz et al. who also observed an association between plasma and CSF ALA and DHA [20]. This data is also consistent with studies in rats showing diets enriched with omega 3 improved brain DHA levels [21]. A positive relationship between the whole blood and CSF omega-6 subfraction AA was also found. While Jumpertz et al. did not observe an association between plasma and CSF omega-6 PUFA’s [20], others have demonstrated that peripherally injected [^11^C]-arachidonic acid can be visualized, after only 15 minutes, in human cranial images [22]. Taken together, this data suggests that in healthy humans PUFA’s can cross the BBB at physiological concentrations. In apparent contrast, Carver et al. reported an inverse association between postmortem human erythrocyte and brain tissue DHA and AA levels [23]. Similarly in schizophrenic patients an inverse association between erythrocyte and CSF DHA levels has been reported [24]. The reasons for these observed inverse associations are unknown, however, it is noted that the specimens used in these studies were either postmortem tissue or from participants with documented neurological abnormalities suggesting the potential for unexpected bias due to either sample quality or clinical condition.

Several models regarding the uptake of fatty acids by the brain have been proposed and recently reviewed by Chen et al. [25]. Current evidence appears to support both transport protein facilitated movement and passive diffusion of fatty acids across the BBB. In 2009 Ouellet et al. demonstrated, using an *in situ* brain perfusion technique in mice, that radiolabeled DHA and eicosapentaenoic acid (EPA; C20:5n-3) readily cross the BBB. As the brain uptake of [^14^C]-DHA and [^14^C]-EPA was not saturable, Ouellet et al. postulated that these compounds enter the brain by passive diffusion [26]. However the presence of several BBB lipid transporter proteins suggests that some fatty acids, at least in part, are facilitated into the CNS. Recently Mitchell et al. investigated fatty acid transport across an *in vitro* BBB model. These researchers demonstrated that fatty acid transport protein 1 and 4 (FATP-1, FATP-4) are the predominant fatty acid transport proteins expressed in the human BBB and that they, in addition to fatty acid translocase/CD36, are involved in fatty acid permeability. It was also observed that the specific chemical structure of individual fatty acids influences the rate of transport, with short to medium chain SFA’s moving across the microvessel monolayer more readily than longer chained SFA’s, whilst unsaturated fatty acids accumulated in the basolateral medium to a higher degree than SFA of similar chain length [27]. If the movement of fatty acids across the BBB is indeed facilitated then the selective uptake of fatty acids by lipid transporter proteins could potentially alter the equilibrium between blood and central lipid pools and may explain the lack of association observed between some respective fatty acid species in this study. Clearly further research into the mechanism of fatty acid transport across the BBB is required.

While it is generally accepted that the brain must obtain essential fatty acids from the blood, evidence suggests that it is also capable of independently synthesizing a variety of lipids [28]. Fatty acids are formed by processes involving the reductive polymerization of acetyl-CoA in which the hydrolysis of ATP provides the energy required for carbon–carbon bond formation. Two main pathways of fatty acid biosynthesis have been described. The first pathway involves the *de novo* synthesis of SFA’s through the action of acetyl-CoA carboxylase and fatty acid synthase. The second pathway involves the desaturation or elongation of these SFA’s by Δ9-desaturase resulting in the formation of (n-5), (n-7) and (n-9) MUFA. The desaturation and elongation of the essential fatty acids, linoleic acid (LA) and ALA, results in the formation of the (n-6) and (n-3) family of PUFA’s respectively [29]. The synthesis of fatty acids is determined by both the cellular demand for fatty acid species, allosteric effectors and the variable availability of substrates. Regulation is often coordinated at both the transcriptional and post-translational level and is influenced by a plethora of factors including circadian rhythms and various nutritional and hormonal stimuli [30,31]. The likely differences in the fatty acid requirement of cells in the periphery compared to those in the CNS combined with the possible variable effects of regulatory mechanisms may explain both the lack of, and unexpected inverse association between some respective fatty acid species in this study.

**Figure 3:**
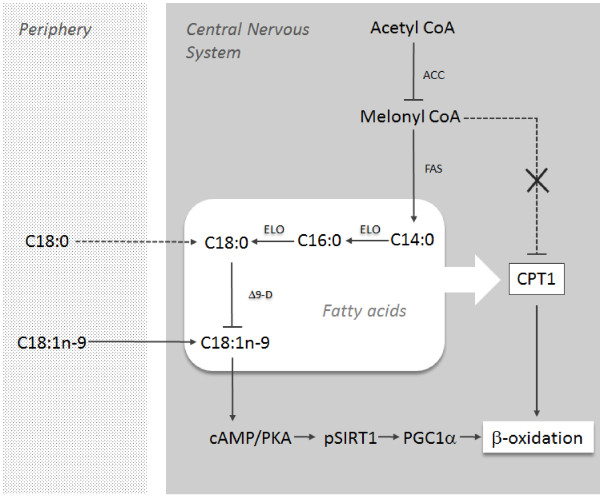
**Interdependence of central stearic and oleic acids.** Due to its unsaturated structure oleic acid (C18:1n-9) moves more readily than stearic acid (C18:0) across the BBB. Within the CNS oleic acid can inhibit both Δ9-D and ACC resulting in decreased *de novo* synthesis of both itself and steric acid. Simultaneously a decrease in melonyl CoA and it’s inhibition of CPT1 can promote an increase in central fatty acid β-oxidation. Oleic acid also increases the expression of genes linked to fatty acid β-oxidation by a SIRT1/PGC1 dependent mechanism. (Abbreviations; ACC, acetyl CoA; FAS, fatty acid synthase; ELO, elongase; Δ9-D, Δ9-desaturase; CPT1, carnitine palmitoyltransferase-1; cAMP/PKA, cyclic adenosine monophosphate/protein kinase A; pSIRT1, phosphorylated sirtuin 1; PGC1α peroxisome proliferator-activated receptor gamma co-activator).

The MUFA oleic acid (C18:1n-9), formed from the desaturation of stearic acid (C18:0) through the activity of Δ9-desaturase, is the primary fatty acid in the white matter of the mammalian brain [32]. In this study an inverse relationship between CSF and whole blood oleic acid was observed. This is consistent with a report by Carver et al. who also observed an inverse association between erythrocyte and brain tissue oleic acid in humans [23]. Importantly oleic acid prevents the *de novo* synthesis of fatty acids by inhibiting the activity of both acetyl CoA carboxylase and Δ9-desaturase [33,34]. Consistent with this action, in this study we found that an increase in whole blood oleic acid was associated with a decrease in the stearic: oleic acid ratio, a putative index of Δ9-desaturase activity. Oleic acid has also been shown to promote fatty acid β-oxidation by reducing malonyl-CoA inhibition of carnitine palmitoyltransferase-1(CPT1) and by increasing the expression of genes linked to β-oxidation by a SIRT1/PGC1α dependent mechanism [35-38]. Though supporting data for the modulatory effect of oleic acid in the CNS specifically in humans is scarce, this may at least partially explain the observed inverse association between whole blood and CSF oleic acid (Figure 3).

In some contrast to our findings, Bourre and Dumont, using rat feeding studies, observed that CNS tissue oleic acid remained constant regardless of dietary intake [39]. The differences in these observations, between rats and humans, may represent species differences for the capacity of local oleic acid production within the CNS.

## Conclusions

To our knowledge this is the first study to provide comparative data on the distribution of fatty acids between the whole blood and CSF of healthy humans. The data presented show a relationship between central and peripheral fatty acids of various degrees of unsaturation and chain length. Evidence that both omega-3 and omega-6 PUFA cross the human BBB at physiological concentrations is provided. As alteration in brain fatty acid proportions has been linked to some neurological disorders, these results provide a preliminary reference point from which deviation from the normal may be gauged. To further elucidate the role of fatty acids in the pathophysiology of neurodegenerative disorders, research into the fatty acid distribution between not only blood and CSF but also brain tissue is required in humans.

One limiting factor of our study is the modest number of matched whole blood and CSF samples. However these samples (CSF with matched blood) do represent essentially healthy individuals with no apparent signs of cognitive decline. It is acknowledged that some samples were from older participants and that ageing results in a number of metabolic changes that could potentially influence fatty acid synthesis and oxidation. Nevertheless, the associations in this analysis were robust to the conservative Bonferonni correction for multiple comparisons and were generally consistent with the limited number of previously published observations.

## Methods

### Participants

Male (n = 4) and female (n = 24) participants, who required a spinal tap for the administration of anesthetic as part of routine care, were recruited at Sydney Adventist Hospital, Australia. The average age of participants was 49 years (SE = 3.6, interquartile range = 34). Participants were excluded from the cohort if they had a confirmed diagnosis of a neurological/neurodegenerative disorder or CNS viral infection. In total 28 CSF and matched blood samples were collected from consenting participants considered in general good health.

Written informed consent was obtained from all participants prior to commencement. Ethical approval was obtained from the Human Research Ethics Committee, Sydney Adventist Hospital.

### Biochemical analysis

Fasting (≥10 h) blood and CSF samples were collected by an accredited anesthetist no longer than 30 minutes apart. CSF samples were collected, prior to injection of spinal anesthetics, via standard lumber puncture. Blood samples were collected into heparinized tubes from an intravenous cannula inserted into a superficial vein on an upper limb, prior to the administration of fluids or anesthetics. CSF samples were prepared by centrifuging at 1800 rpm for 10 minutes. All samples were stored in butylated hydroxyl toluene coated tubes, within 1 hour of collection, at −194 degrees Celsius until analysis.

The fatty acid composition of whole blood and CSF lipids was determined according to a modification in the method of Lepage and Roy [40], using an acetyl chloride methylation procedure. Briefly 2 ml of methanol:toluene (4:1 v/v) containing C19:0 (4 μg/ml) internal standard was added to 200 μl of whole blood and 500 μl of CSF. The fatty acids were then methylated by adding 200 μl of acetyl chloride and heated at 100 degrees Celsius for 1 hour. The reaction was then stopped by adding 5 ml of 6% K_2_CO_3_ solution. The samples were centrifuged at 3000 x g for 10 minutes and the supernatant containing the fatty acid methyl esters was transferred into glass vials.

Fatty acid methyl esters were quantified using a Hewlett Packard 6890 Gas Chromatograph equipped with a flame ionization detector. The identity of each fatty acid peak in the sample was determined by comparing the peaks retention time with the retention time of synthetic standards of known fatty acid composition. Fatty acid results are reported as percentage of total fatty acids.

### Statistical analysis

Statistical analyses were performed using SPSS version 16.0 for Windows. Data in Table 1 is presented as means ± standard error. The minimum and maximum observed value is also shown. Multiple linear regression, controlling for age, was used to identify significant relationships between whole blood and CSF fatty acids. Levene’s Test of Equality was used to check homogeneity of variances between groups while both the Kolmogorov-Smirnov, Shapiro-Wilk and histogram analysis was used to check normality of the variables. If the variances of the groups were found to be either not homogenous and/or normality tests for the variables were significant then further investigation with graphical displays was performed to assess the distributions of the variables. Adjusted P-values are provided throughout with test significance set at P-value < 0.05 after Bonferroni correction.

## Abbreviations

ALA: Alpha-linolenic acid; AA: Arachidonic acid; BBB: Blood brain barrier; CPT1: Carnitine palmitoyltransferase-1; CSF: Cerbrospinal fluid; cAMP/PKA: Cyclic adenosine monophosphate/protein kinase A; Δ9-D: Delta 9-desaturase; DHA: Docosohexanoic acid; DPA: Docosopentanoic acid; ELO: Elongase; EPA: Ecosopentanoic acid; FAS: Fatty acid synthase; FATP-1, FATP-4: Fatty acid transport protein 1 and 4; MFA: Monounsaturated fatty acid; PGC1α: Peroxisome proliferator-activated receptor gamma co-activator; pSIRT1: Phosphorylated Sirtuin 1; PUFA: Polyunsaturated fatty acid; SFA: Saturated fatty acid.

## Competing interests

The authors declare that they have no competing interests.

## Authors’ contributions

JG/RG planned experiments, recruited subjects, interpreted data, wrote manuscript. JG/MG analysed samples, helped interpret data and reviewed manuscript. AB provided expert statistical analysis of data and reviewed manuscript. All authors read and approved the final manuscript.
